# Magnetic resonance imaging and ultrasound for prediction of residual tumor size in early breast cancer within the ADAPT subtrials

**DOI:** 10.1186/s13058-021-01413-y

**Published:** 2021-03-18

**Authors:** Monika Graeser, Simone Schrading, Oleg Gluz, Kevin Strobel, Christopher Herzog, Lale Umutlu, Alex Frydrychowicz, Dorothea Rjosk-Dendorfer, Rachel Würstlein, Ralph Culemann, Christine Eulenburg, Jascha Adams, Henrik Nitzsche, Anna Prange, Sherko Kümmel, Eva-Maria Grischke, Helmut Forstbauer, Michael Braun, Jochem Potenberg, Raquel von Schumann, Bahriye Aktas, Cornelia Kolberg-Liedtke, Nadia Harbeck, Christiane K. Kuhl, Ulrike Nitz

**Affiliations:** 1 West German Study Group, Ludwig-Weber-Strasse 15, 41061 Moenchengladbach, Germany; 2 Ev. Hospital Bethesda, Breast Center Niederrhein, Ludwig-Weber-Strasse 15, 41061 Moenchengladbach, Germany; 3 Department of Gynecology, University Medical Center Hamburg, Martinistrasse 52, 20251 Hamburg, Germany; 4 Department of Diagnostic and Interventional Radiology, Hospital of the University of Aachen, RWTH, Pauwelsstrasse 30, 52074 Aachen, Germany; 5 University Hospital Cologne, Kerpener Strasse 62, 50937 Cologne, Germany; 6 Radiology, Burgstrasse 7, 80331 Munich, Germany; 7 Department of Diagnostic and Interventional Radiology and Neuroradiology, University Hospital Essen, University Duisburg-Essen, Hufelandstrasse 55, 45147 Essen, Germany; 8 Department of Radiology and Nuclear Medicine, Schleswig-Holstein University Hospital, Campus Lübeck, Ratzeburger Allee 160, 23562 Lübeck, Germany; 9 Department of Radiology, University Hospital, LMU Munich, Marchioninistrasse. 15, 81377 Munich, Germany; 10 Department of Gynecology and Obstetrics, Breast Center, University of Munich (LMU) and CCCLMU, Marchioninistrasse 15, 81377 Munich, Germany; 11 Medizinisches Versorgungszentrum Radiologie Rhein-Sieg, GFO Kliniken Troisdorf, Hospitalstrasse 45, 53840 Troisdorf, Germany; 12 Alcedis GmbH, Winchesterstrasse 3, 35394 Giessen, Germany; 13 Department of Radiology, Clinics Essen-Mitte, Breast Centre, Henricistrasse 92, 45136 Essen, Germany; 14 Clinics Essen-Mitte, Breast Centre, Henricistrasse 92, 45136 Essen, Germany; 15 University Hospital Charité, Women’s Clinic, Berlin, Charitéplatz 1, 10117 Berlin, Germany; 16 University Clinic Tuebingen, Women’s Clinic, Calwerstrasse 7, 72076 Tuebingen, Germany; 17 Practice Network Troisdorf, Schlossstrasse 18, 53840 Troisdorf, Germany; 18 Red Cross Women’s Hospital, Nymphenburger Strasse 163, 80634 Munich, Germany; 19 Ev. Waldkrankenhaus Berlin, Stadtrandstrasse 555, 13589 Berlin, Germany; 20 Department of Gynecology and Obstetrics, University Hospital Essen, Hufelandstrasse 55, 45147 Essen, Germany; 21 Department of Gynecology, University Hospital Leipzig, Liebeigstrasse 20A, 04103 Leipzig, Germany

**Keywords:** Breast cancer, Neoadjuvant therapy, Magnetic resonance imaging, Ultrasound, Residual tumor size

## Abstract

**Background:**

Prediction of histological tumor size by post-neoadjuvant therapy (NAT) ultrasound and magnetic resonance imaging (MRI) was evaluated in different breast cancer subtypes.

**Methods:**

Imaging was performed after 12-week NAT in patients enrolled into three neoadjuvant WSG ADAPT subtrials. Imaging performance was analyzed for prediction of residual tumor measuring ≤10 mm and summarized using positive (PPV) and negative (NPV) predictive values.

**Results:**

A total of 248 and 588 patients had MRI and ultrasound, respectively. Tumor size was over- or underestimated by < 10 mm in 4.4% and 21.8% of patients by MRI and in 10.2% and 15.8% by ultrasound. Overall, NPV (proportion of correctly predicted tumor size ≤10 mm) of MRI and ultrasound was 0.92 and 0.83; PPV (correctly predicted tumor size > 10 mm) was 0.52 and 0.61. MRI demonstrated a higher NPV and lower PPV than ultrasound in hormone receptor (HR)-positive/human epidermal growth factor receptor 2 (HER2)-positive and in HR−/HER2+ tumors. Both methods had a comparable NPV and PPV in HR−/HER2− tumors.

**Conclusions:**

In HR+/HER2+ and HR−/HER2+ breast cancer, MRI is less likely than ultrasound to underestimate while ultrasound is associated with a lower risk to overestimate tumor size. These findings may help to select the most optimal imaging approach for planning surgery after NAT.

**Trial registration:**

Clinicaltrials.gov, NCT01815242 (registered on March 21, 2013), NCT01817452 (registered on March 25, 2013), and NCT01779206 (registered on January 30, 2013).

**Supplementary Information:**

The online version contains supplementary material available at 10.1186/s13058-021-01413-y.

## Background

Neoadjuvant therapy (NAT) allows monitoring of tumor response to treatment, provides important prognostic information, and may permit breast-conserving surgery by downstaging cancer [1]. Efficacy of NAT can be measured using pathological complete response (pCR), most commonly defined as the absence of invasive cancer and in situ cancer in the breast and axillary nodes (ypT0 ypN0); absence of invasive cancer in the breast and axillary nodes, irrespective of ductal carcinoma in situ (ypT0/is ypN0); and absence of invasive cancer in the breast irrespective of ductal carcinoma in situ or nodal involvement (ypT0/is). A meta-analysis by Cortazar et al. demonstrated that pCR defined as either ypT0/is ypN0 or ypT0 ypN0 was associated with improved overall survival and event-free survival compared to ypT0/is without information on the nodal status. Nowadays, ultrasound (US) and magnetic resonance imaging (MRI) are commonly used to monitor tumor response to NAT, and several studies have investigated their application for the prediction of pCR and measurement of residual tumor. Assessment of tumor size after NAT allows to determine the best surgical approach, or alternatively, it may provide evidence to continue the same systemic therapy or switch to another regimen [2, 3]. However, there is conflicting evidence regarding the accuracy of these two methods for the evaluation of residual tumors with some studies indicating the superiority of MRI [4, 5] while other reports suggest comparable accuracy of MRI and US [6–8]. The accuracy of the employed method in estimating tumor size has a profound impact on the success of surgery in terms of long-term outcomes and good cosmetic results. Only the most precise evaluation of the lesion allows complete tumor resection while keeping the removal of surrounding tissue to a minimum. Underestimation of lesion size carries the risk of resection with tumor-positive margins thus potentially worsening patient prognosis and requiring repeat surgery. Overestimation, however, may increase the likelihood of mastectomy in cases where breast-conserving surgery would have normally been recommended [9]. Therefore, the accuracy of the imaging method is of profound importance for disease prognosis and for patients’ quality of life. Thus far, only a few studies have compared the post-NAT assessment of tumor size by MRI and US with histological measurements as the gold standard.

The present imaging subproject was performed within the framework of the Adjuvant Dynamic Marker-Adjusted Personalized Therapy Trial Optimizing Risk Assessment and Therapy Response Prediction in Early Breast Cancer (ADAPT) umbrella trial conducted by the West German Study Group (WSG). The aim of the ADAPT study was to identify early markers for therapy response to individualize NAT by avoiding over- and under-treatment. The primary objective of this analysis was to compare the value of MRI versus US performed at the end of neoadjuvant therapy (EoT) for prediction of pCR and residual disease and their accuracy in predicting histological tumor size in hormone receptor-positive, human epidermal growth factor receptor-2 positive (HR+/HER2+), HR−/HER2−, and HR−/HER2+ tumors from the respective ADAPT substudies.

## Methods

### Study design

Details on the design of WSG-ADAPT, a prospective, controlled, randomized, non-blinded, multi-center, and investigator-initiated umbrella clinical trial, and the results of three substudies, ADAPT triple-negative (TN, NCT01815242), ADAPT HER2+/HR+ (NCT01817452), and ADAPT HER2+/HR− substudies (NCT01779206), have been previously reported [10–13]. With regard to this analysis, three breast cancer subtypes were investigated: (i) HR+/HER2+ tumors after NAT including trastuzumab emtansine monotherapy (T-DM1), T-DM1+endocrine therapy (ET), or trastuzumab+ET; (ii) HR−/HER2− tumors after neoadjuvant nab-paclitaxel+gemcitabine or nab-paclitaxel+carboplatin; and (iii) HR−/HER2+ tumors after neoadjuvant trastuzumab+pertuzumab treatment with or without paclitaxel. Enrolled patients were examined with US (mandatory) and MRI (optional) before systemic therapy, at 3 and 6 weeks after the start of NAT and at EoT. Here, we investigated only those patients with MRI and/or US performed at EoT. After neoadjuvant therapy, surgery within 3 weeks or histologic confirmation of non-pCR by core needle biopsy was obligatory. In case of a histologically confirmed residual invasive tumor by core needle biopsy, patients received standard NAT according to the national guidelines and underwent surgery afterwards. Clinically node-positive patients underwent axillary dissection after completion of NAT. Sentinel node biopsy in clinically node-negative patients was performed either before or after NAT, at the investigator’s discretion. Adjuvant therapy was administered according to the national guidelines.

### Eligibility criteria for enrollment of the patients

Women aged ≥18 years, with histologically confirmed unilateral, primary invasive BC, and HR/HER2 status centrally confirmed at Institute of Pathology, University of Hannover, Germany, were eligible to participate in the study. HR-positive (≥1% of tumor nuclei staining positive for estrogen receptor and/or progesterone receptor) and HER2-positive status (immunohistochemistry (IHC) 3+ positive or in situ hybridization (ISH) positive) was required for participation in the ADAPT HER2+/HR+ substudy, HR-negative (< 1% of tumor nuclei staining positive for estrogen receptor and progesterone receptor) and HER2-positive status was required for the ADAPT HER2+/HR− substudy, and HR-negative and HER2-negative (IHC 1+ negative or IHC 0 negative, or ISH negative) status was required for participation in the ADAPT TN substudy. Eastern Cooperative Oncology Group Performance Status ≤1 or Karnofsky Performance Status ≥80%, normal organ function, and adequate hematologic parameters were required for inclusion. Detailed inclusion and exclusion criteria for participation in the ADAPT study have been described elsewhere [10–13]. All patients provided written informed consent prior to study enrollment.

### MRI technique

Breast MRI was performed at 43 sites thus providing an overview regarding radiology practice in Germany. In order to obtain images of comparable quality across all study locations, the central MRI reading site (Department of Diagnostic and Interventional Radiology, University Hospital, RWTH Aachen, Germany) provided a standardized imaging protocol which consisted of basic sequences; there was no need for special hardware or software. Prior to study participation, images provided by local reading sites were evaluated by the central reading site to ensure that the required MRI technique standards were met.

The standardized imaging protocol of 1.5-T and 3.0-T systems with a dedicated breast multichannel surface coil consisted of an axial bilateral two-dimensional multi-section gradient-echo dynamic series (repetition time 250 ms; echo time 4.6 ms (1.5 T) or 2.3 ms (3 T); flip angle 90°) with a section thickness of 3 mm and full 512 × 512 acquisition matrix. The voxel size of all scans was kept constant along all exams with a maximum of 0.8. To ensure a high spatial resolution, the field of view was adapted to the individual breast size of the patient with a minimum of 280 mm and a maximum of 350 mm keeping the voxel size within the intended range. The dynamic sequence was performed prior to and four times after bolus injection of macrocyclic gadolinium agent, gadobutrol (Gadovist®/Gadavist®, Bayer AG, Leverkusen, Germany; 0.1 mmol/kg body weight), followed by a saline flush. Depending on the site preference, fat suppression or image subtraction was used for visualization of enhancement in the T1 gradient-echo sequence. A standard center of *k*-space between 60 and 90 s was used in all exams. In addition, an axial T2-weighted fast spin-echo sequence without fat suppression and with identical anatomic parameters as the T1-gradient echo sequence was performed.

### MRI interpretation

A blinded analysis of locally acquired MRI images was conducted at the central reading site by two specialized breast radiologists with 13 (SS) and 23 years (CK) of experience in interpreting breast MRI images. Images were read according to BI-RADS guidelines (5th edition, [14]). Initially, the first (early) post-contrast subtracted or fat-suppressed T1 image was read for an overview of enhancing lesions and to access background enhancement. Afterwards, the complete unsubtracted dynamic series pre- and post-contrast at each slice was thoroughly analyzed for characteristics of any enhancing lesion and evaluation of enhancing residual disease. Then, the T2-weighted series was analyzed for any structural changes and fluid containing lesions/edema. Any lesions in the dynamic contrast-enhanced images were correlated with the T2-weighted series for morphology and signal intensity. Final evaluation for complete imaging response was based on the whole dynamic series, at which enhancement characteristic and morphologic criteria at T1 pre- and post-contrast considering the early and late phase and also on the signal intensity on T2. Lesion size was measured in the longest diameter on the unsubtracted images. In patients with no visible tumor after NAT, anatomic landmarks in non-subtracted T1- and T2-weighted images were analyzed to identify the site of the lesion.

### US imaging

Before the first NAT cycle, patients underwent a systematic sonographic examination of both breasts and axillae by experienced gynecologists using breast US systems with at least 7.5 MHz and an electronic linear US probe. If possible, the tumor was measured in one to three diameters, and measurements were registered in the electronic case report forms. US was then repeated after one and two cycles of NAT and at EoT. All US images were read and interpreted at the local study site by experienced gynecologists. The lesions were described, and tumor size measured. The tumor was marked with a clip before the first cycle of NAT to be able to reliably identify the tumor region at the subsequent examinations.

### Histological assessment

Post-NAT surgical specimens underwent local histopathological assessment, and the longest diameter of the residual tumor was documented. No histopathological evidence of residual invasive tumor cells, either in the breast or the axillary lymph nodes (ypT0/is ypN0), was denoted as pCR.

### Statistical analyses

Accuracy of post-NAT US and MRI was analyzed for prediction of residual tumor size of ≥10 mm (the gold standard). The concordance between imaging and histological assessment was summarized by computing Spearman’s correlation coefficient, graphically displaying the difference in tumor diameter (mm) between imaging and histological assessment, as well as comparing the positive predictive value (PPV), negative predictive value (NPV), sensitivity, and specificity in each tumor subtype. PPV was defined as the proportion of correctly predicted tumors measuring > 10 mm on post-NAT imaging compared to the final pathology specimen. NPV was defined as the proportion of correctly predicted tumors measuring ≤10 mm by imaging. In addition, we analyzed the value of US and MRI for the prediction of pCR by calculating PPV (probability that non-pCR was documented when no complete response was observed), NPV (probability that pCR was actually achieved following complete response on imaging), sensitivity, and specificity. Receiver operating characteristic (ROC) curves and ROC area under the curve (AUC) predicting a residual tumor from imaging were computed. Furthermore, patient characteristics at baseline were compared between the cohorts with tumor size overestimation or underestimation by imaging (difference > 10 mm for both) or with a ≤ 10 mm difference in tumor size between imaging and histological assessments.

Available data were analyzed and compared between the three groups: patients who underwent MRI (MRI group), patients who underwent US (US group), and patients who underwent both MRI and US (MRI and US group).

Statistical data analyses were performed with SAS software (version 9.4, SAS Institute, NC) and Stata (version 16.1/ SE, StataCorp LLC, TX). Graphs, including the quadratic curves, were plotted using the GraphPad Prism 8 software (GraphPad Software, CA).

## Results

### Patient characteristics

**Fig. 1:**
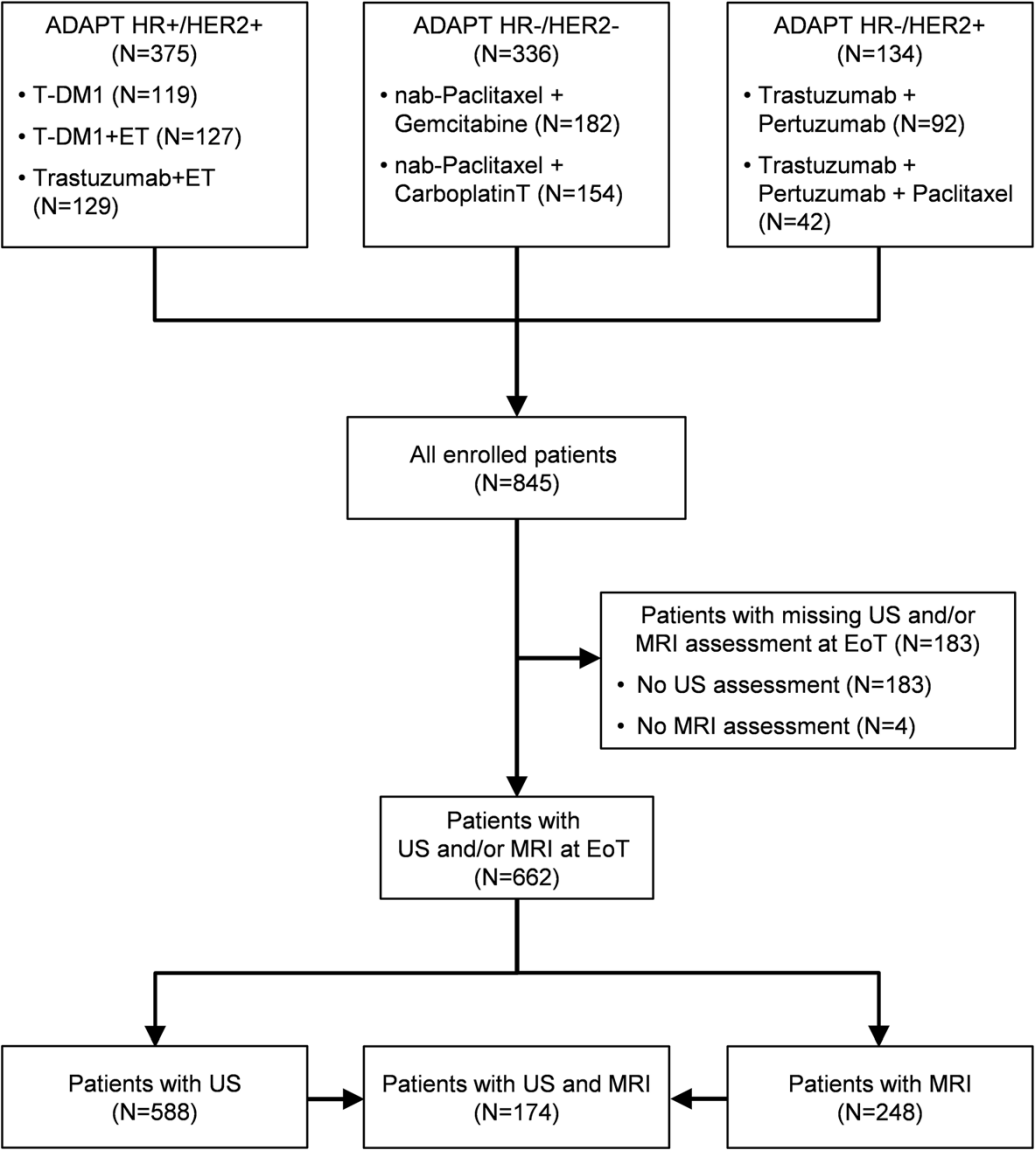
CONSORT diagram. EoT, end of therapy; ET, endocrine therapy; MRI, magnetic resonance imaging, pCR, pathological complete response; T-DM1, trastuzumab emtansine; US, ultrasound

A total of 845 patients at 58 sites in Germany were enrolled from October 2012 to December 2015, of which 375 were randomized into the ADAPT HR+/HER2+ substudy, 336 into the ADAPT HR−/HER2− substudy, and 134 into the ADAPT HR−/HER2+ substudy (Fig. 1). A total of 662 patients were investigated with MRI and/or US at EoT, of whom 588 patients had US, 248 had MRI, and 174 had both imaging techniques. Data on tumor length were available in 229/248 MRI measurements, 577/588 US measurements, and in 808/845 pathological evaluations (243/248 within the MRI group, 571 within the US group, and 169/174 within the group of both US and MRI evaluations).

The baseline characteristics were comparable between patients from the MRI group (Table 1) and the US group (Table 2) with a median age of 52 and 51 years, rates of T1 at 44.0% and 40.1%, T2 at 51.2% and 53.6%, N0 at 66.1% and 70.4%, and N1 at 29.4% and 26.2%, respectively.

**Table 1:** Patient characteristics (MRI group) according to tumor size estimation and discordance between MRI and histology

	Overestimation MRI – Histol. > 10 mm	Difference MRI – Histol. ≤10 mm	Underestimation MRI – Histol. > 10 mm	Unknown	Total
Number of patients (% of total)	11 (4.44)	159 (64.11)	54 (21.77)	24 (9.68)	248 (100.00)
Age at initial visit [years]					
Mean	43.27	52.06	52.19	49.33	51.43
SD	6.18	11.31	11.29	9.47	11.08
Median	44.00	52.00	53.00	50.00	52.00
Min	26.00	25.00	30.00	34.00	25.00
Max	50.00	78.00	75.00	70.00	78.00
N.d.	0	2	0	0	2
Central grade, *N* (%)					
1	0 (0.00)	4 (80.00)	1 (20.00)	0 (0.00)	5 (100.00)
2	5 (4.49)	66 (65.35)	22 (21.78)	8 (7.92)	101 (100.00)
3	6 (4.29)	88 (62.86)	30 (21.43)	16 (11.43)	140 (100.00)
N.d.	0 (0.00)	1 (50.00)	1 (50.00)	0 (0.00)	2 (100.00)
Clinical baseline characteristics, *N* (%)					
cT					
1	5 (4.59)	80 (73.39)	19 (17.43)	5 (4.59)	109 (100.00)
2	6 (4.72)	78 (61.42)	29 (22.83)	14 (11.02)	127 (100.00)
3	0 (0.00)	1 (10.00)	5 (50.00)	4 (40.00)	10 (100.00)
4	0 (0.00)	0 (0.00)	1 (50.00)	1 (50.00)	2 (100.00)
cN					
0	6 (3.66)	121 (73.78)	30 (18.29)	7 (4.27)	164 (100.00)
1	5 (6.85)	34 (46.58)	21 (28.77)	13 (17.81)	73 (100.00)
2	0 (0.00)	4 (40.00)	3 (30.00)	3 (30.00)	10 (100.00)
3	0 (0.00)	0 (0.00)	0 (0.00)	1 (100.00)	1 (100.00)
Menopausal status, *N* (%)					
Premenopausal	11 (9.24)	72 (60.50)	23 (19.84)	13 (10.92)	119 (100.00)
Postmenopausal	0 (0.00)	80 (67.23)	28 (23.53)	11 (9.24)	119 (100.00)
Unknown/unclear	0 (0.00)	7 (70.00)	3 (30.00)	0 (0.00)	10 (100.00)
BC subtype, *N* (%)					
HR+/HER2+	5 (4.72)	66 (62.26)	30 (28.30)	5 (4.72)	106 (100.00)
HR−/HER2−	6 (6.52)	62 (67.39)	13 (14.13)	11 (11.96)	92 (100.00)
HR−/HER2+	0 (0.00)	31 (62.00)	11 (22.00)	8 (16.00)	50 (100.00)

**Table 2:** Patient characteristics (US group) according to tumor size estimation and discordance between US and histology

	Overestimation US – Histol. > 10 mm	Difference US – Histol. ≤10 mm	Underestimation US – Histol. > 10 mm	Unknown	Total
Number of patients (% of total)	60 (10.20)	407 (69.22)	93 (15.82)	28 (4.76)	588 (100.00)
Age at initial visit [years]					
Mean	53.13	51.16	52.89	52.46	51.70
SD	12.49	11.71	9.54	10.84	11.44
Median	50.00	51.00	53.00	50.00	51.00
Min	31.00	21.00	32.00	30.00	21.00
Max	75.00	77.00	78.00	74.00	78.00
N.d.	0	2	0	0	2
Central grade, *N* (%)					
1	0 (0.00)	8 (80.00)	2 (20.00)	0 (0.00)	10 (100.00)
2	32 (13.60)	168 (71.19)	27 (11.44)	9 (3.81)	236 (100.00)
3	28 (8.26)	228 (67.26)	64 (18.88)	19 (5.60)	339 (100.00)
N.d.	0 (0.00)	3 (100.00)	0 (0.00)	0 (0.00)	3 (100.00)
Clinical baseline characteristics, *N* (%)					
cT					
1	15 (6.36)	196 (83.05)	14 (5.93)	11 (46.61)	236 (100.00)
2	35 (11.11)	196 (62.22)	68 (21.59)	16 (5.08)	315 (100.00)
3	7 (23.33)	12 (40.00)	10 (33.33)	1 (3.33)	30 (100.00)
4	3 (42.86)	3 (42.86)	1 (14.29)	0 (0.00)	7 (100.00)
cN					
0	33 (7.97)	304 (73.43)	58 (14.01)	19 (4.59)	414 (100.00)
1	25 (16.23)	93 (60.39)	30 (19.48)	6 (3.90)	154 (100.00)
2	2 (10.53)	9 (47.37)	5 (26.32)	3 (15.79)	19 (100.00)
3	0 (0.00)	1 (100.00)	0 (0.00)	0 (0.00)	1 (100.00)
Menopausal status, *N* (%)					
Premenopausal	31 (10.99)	199 (70.57)	38 (13.48)	14 (4.96)	282 (100.00)
Postmenopausal	28 (10.07)	189 (67.99)	49 (17.63)	12 (4.32)	278 (100.00)
Unknown/unclear	1 (3.57)	19 (67.86)	6 (21.43)	2 (7.14)	28 (100.00)
BC subtype, *N* (%)					
HR+/HER2+	28 (10.98)	187 (73.33)	31 (12.16)	9 (3.53)	255 (100.00)
HR−/HER2−	26 (10.44)	166 (66.67)	45 (18.07)	12 (4.82)	249 (100.00)
HR−/HER2+	6 (7.14)	54 (64.29)	17 (20.24)	7 (8.33)	84 (100.00)

In the MRI group, the difference in tumor size between MRI and histology was ≤10 mm in 64.1% of patients (*n* = 159/248, Table 1). Histological tumor size was overestimated by MRI by > 10 mm only in 4.4% of patients (*n* = 11/248) and underestimated by > 10 mm in 21.8% of patients (*n* = 54/248). There were no HR−/HER2+ tumors with size overestimation by MRI, whereas tumor size was underestimated in 22% of patients with this BC subtype (*n* = 11/50). Tumor size was underestimated in only 14.1% of HR−/HER2− (*n* = 13/92) and in 28.3% of HR+/HER2+ tumors (*n* = 30/106).

In the US group, estimated tumor size differed from histology by ≤10 mm in 69.2% of patients (*n* = 407/588, Table 2). US overestimated and underestimated by > 10 mm in 10.2% (*n* = 60) and 15.8% of patients (*n* = 93), respectively. A similar proportion of HR+/HER2+ tumors had tumor size over- and underestimation (11%, *n* = 28/255 and 12.2%, *n* = 31). However, underestimation by US was more frequent than overestimation in the HR−/HER2− (18.1%, *n* = 45/249, vs 10.4%, *n* = 26) and in the HR−/HER2+ group (20.2%, *n* = 17/84, vs 7.1%, n = 6/84).

### Prediction of pCR and presence of residual tumor

Data on pCR was available in 815 patients (in 244/248 patients in the MRI group, in 578/588 patients in the US group, and in 171/174 patients with MRI and US). pCR was achieved in 36.5% of patients in the MRI (*n* = 89/244), 37.7% in the US group (*n* = 218/578), and 37.4% (*n* = 64/171) in the MRI and US group. The sensitivity of US and MRI was similar overall and in HR+/HER2+ tumors while sensitivity was higher for US in HR−/HER2− tumors (0.89 vs 0.73 for MRI) and higher for MRI in HR−/HER2+ tumors (0.96 vs 0.81 for US, Table 3). MRI yielded a higher specificity than US in all tumors (0.69 vs 0.46) and in HR+/HER2+ (0.56 vs 0.47), HR−/HER2− (0.71 vs 0.52), and HR−/HER2+ tumors (0.83 vs 0.33). Overall, NPV was similar between MRI and US; however, MRI had a higher NPV in HR+/HER2+ (0.76 vs 0.63) and HR−/HER2+ tumors (0.95 vs 0.64), while US had a higher NPV in HR−/HER2− tumors (0.74 vs 0.58). MRI had a higher PPV than US in all tumors (0.83 vs 0.73) and in HR−/HER2+ BC (0.86 vs 0.55) while both methods had a similar PPV in HR+/HER2+ and HR−/HER2− tumors.

**Table 3:** Prediction of pCR and residual tumor size in the MRI group and in the US group

**Group**		**pCR,** ***N***	**CR,** ***N***	**TN,** ***N***	**FP,** ***N***	**TP,** ***N***	**FN,** ***N***	**Specificity (CL)**	**Sensitivity (CL)**	**NPV (CL)**	**PPV (CL)**
**Overall**	**MRI (*N*** **= 244)**	89	84	61	28	132	23	0.69 (0.58–0.78)	0.85 (0.79–0.90)	0.73 (0.62–0.82)	0.83 (0.76–0.88)
	**US (*N*** **= 578)**	218	149	101	117	312	48	0.46 (0.40–0.53)	0.87 (0.83–0.90)	0.68 (0.60–0.75)	0.73 (0.68–0.77)
**HR+/HER2+**	**MRI (*N*** **= 103)**	34	25	19	15	63	6	0.56 (0.38–0.73)	0.91 (0.82–0.97)	0.76 (0.55–0.91)	0.81 (0.70–0.89)
	**US (*N*** **= 252)**	83	62	39	44	146	23	0.47 (0.36–0.58)	0.86 (0.80–0.91)	0.63 (0.50–0.75)	0.77 (0.70–0.83)
**HR−/HER2−**	**MRI (*N*** **= 91)**	31	38	22	9	44	15	0.71 (0.52–0.86)	0.73 (0.60–0.84)	0.58 (0.41–0.74)	0.83 (0.70–0.92)
	**US (*N*** **= 242)**	93	65	48	45	132	17	0.52 (0.41–0.62)	0.89 (0.82–0.93)	0.74 (0.62–0.84)	0.75 (0.68–0.81)
**HR−/HER2+**	**MRI (*N*** **= 50)**	24	21	20	4	25	1	0.83 (0.63–0.95)	0.96 (0.80–1)	0.95 (0.76–1)	0.86 (0.68–0.96)
	**US (*N*** **= 84)**	42	22	14	28	34	8	0.33 (0.20–0.50)	0.81 (0.66–0.91)	0.64 (0.41–0.83)	0.55 (0.42–0.68)
**Group**		**≤10 mm at histology,** ***N***	**≤10 mm at imaging,** ***N***	**TN,** ***N***	**FP,** ***N***	**TP,** ***N***	**FN,** ***N***	**Specificity (CL)**	**Sensitivity (CL)**	**NPV (CL)**	**PPV (CL)**
**Overall**	**MRI (*N*** **= 224)**	151	100	92	59	65	8	0.61 (0.53–0.69)	0.89 (0.80–0.95)	0.92 (0.85–0.96)	0.52 (0.43–0.62)
	**US (*N*** **= 560)**	353	306	253	100	154	53	0.72 (0.67–0.76)	0.74 (0.68–0.80)	0.83 (0.78–0.87)	0.61 (0.54–0.67)
**HR+/HER2+**	**MRI (*N*** **= 101)**	61	30	27	34	37	3	0.44 (0.32–0.58)	0.93 (0.80–0.98)	0.90 (0.74–0.98)	0.52 (0.40–0.64)
	**US (*N*** **= 246)**	142	138	106	36	72	32	0.75 (0.67–0.82)	0.69 (0.59–0.78)	0.77 (0.69–0.84)	0.67 (0.57–0.75)
**HR−/HER2−**	**MRI (*N*** **= 81)**	55	45	40	15	21	5	0.73 (0.59–0.84)	0.81 (0.61–0.93)	0.89 (0.76–0.96)	0.58 (0.41–0.74)
	**US (*N*** **= 237)**	156	124	107	49	64	17	0.69 (0.61–0.76)	0.79 (0.69–0.87)	0.86 (0.79–0.92)	0.57 (0.47–0.66)
**HR−/HER2+**	**MRI (*N*** **= 42)**	35	25	25	10	7	0	0.71 (0.54–0.85)	1 (0.59–1)	1 (0.86–1)	0.41 (0.18–0.67)
	**US (*N*** **= 77)**	55	44	40	15	18	4	0.73 (0.59–0.84)	0.82 (0.60–0.95)	0.91 (0.78–0.98)	0.55 (0.36–0.72)

*TP*, true-positive examination; *FP*, false-positive examination; *TN*, true-negative examination and *FN*; false-negative examination. For pCR, TN was defined as CR at imaging and pCR, FP as no CR at imaging and pCR, TP as no CR at imaging and no pCR, and FN as CR at imaging and no pCR. For residual tumor at histology, TN was defined as tumor size of ≤10 mm by imaging and equivalent tumor size at histology, FP was defined as tumor size of > 10 mm by imaging and tumor size of ≤10 mm at histology; TP was defined as tumor size of > 10 mm by imaging and equivalent tumor size at histology, and FN was defined as tumor size of ≤10 mm by imaging and tumor size of > 10 mm at histology. *PPV*, positive predictive value, defined as *P*(*P* = 1|*I* = 1); *NPV*, negative predictive value, defined as *P*(*P* = 0|*I* = 0); *SENS*, sensitivity, defined as *P*(*I* = 1|*P* = 1); *SPEC*, specificity, defined as *P*(*I* = 0|*P* = 0 where *P*(A|B) denotes the conditional probability of event A given that event B has occurred. *P* = 1 denotes a residual invasive tumor size of > 10 mm at histology, and *I* = 1 denotes tumor size of > 10 mm at imaging; CL, exact 95% confidence limits (Clopper-Pearson)

**Fig. 2:**
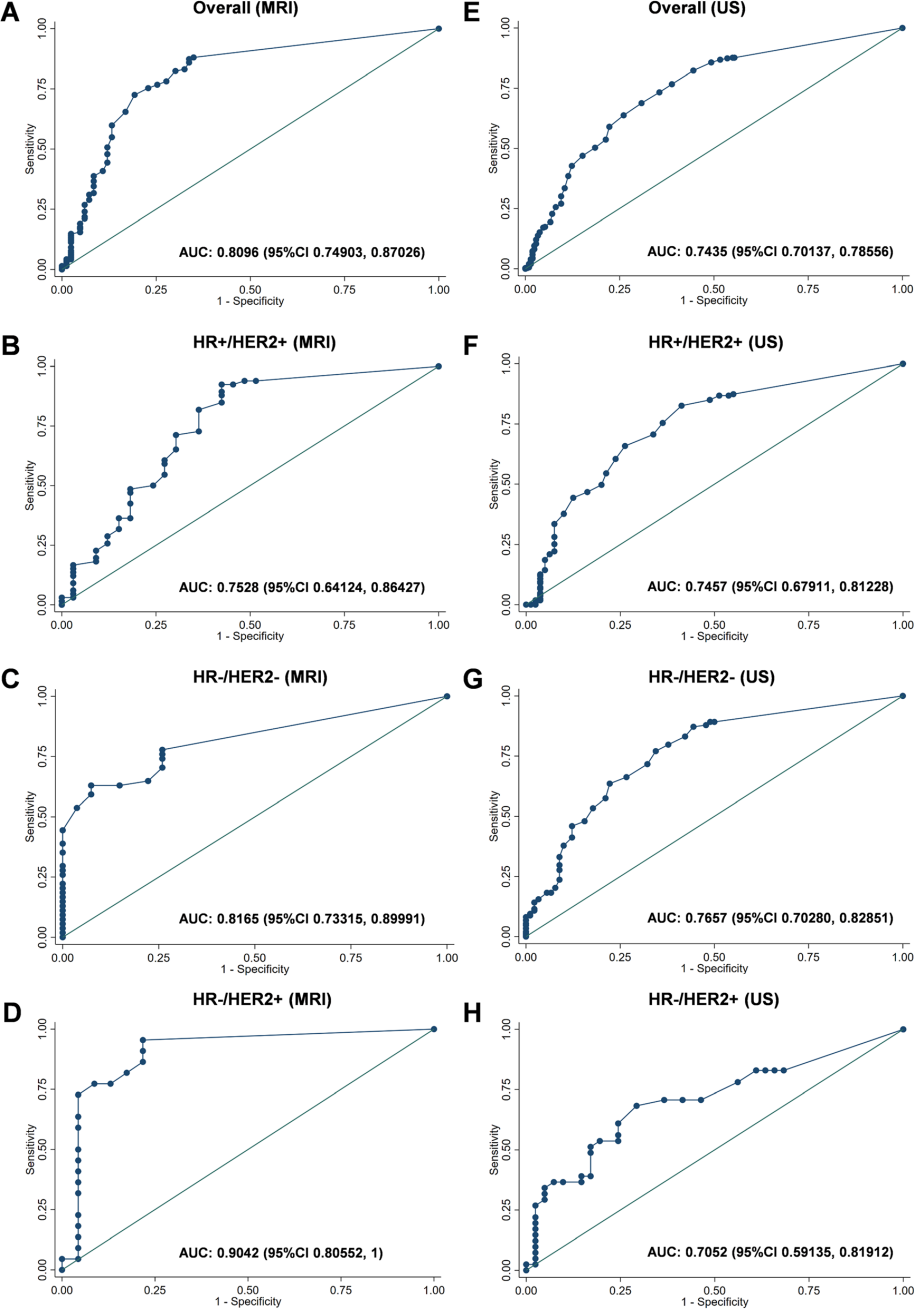
ROC curves for detecting pCR by MRI (**a**–**d**) and by US (**e**–**h**). Data are shown for all tumors (**a**, **e**) and for patients with HR+/HER2+ (**b**, **f**), HR−/HER2− (**c**, **g**), and HR−/HER2+ (**d**, **h**) tumors

In the ROC analysis, MRI demonstrated a larger AUC than US in all tumors (0.80 vs 0.74) and in HR−/HER2+ BC (0.90 vs 0.71, Fig. 2). AUC was similar for MRI and US in HR+/HER2+ (0.75, both) and HR−/HER2− tumors (0.82 and 0.77). Comparable predictive values were obtained in the MRI and US group (Supplementary Table 1 and Supplementary Figure 1).

### Prediction of residual tumor size > 10 mm by MRI and US

US yielded a higher specificity than MRI overall (0.72 vs 0.61) and in HR+/HER2+ tumors (0.75 vs 0.44) while both methods were comparable in HR−/HER2− and HR−/HER2+ BC (Table 3). MRI demonstrated a higher sensitivity and NPV than US among all tumors (sensitivity 0.89 vs 0.74; NPV 0.92 vs 0.83) and in HR+/HER2+ (sensitivity 0.93 vs 0.69; NPV 0.9 vs 0.77) and HR−/HER2+ BC (sensitivity 1 vs 0.82; NPV 1 vs 0.91). MRI and US had similar sensitivity and NPV in HR−/HER2− tumors. PPV was higher for US than for MRI in HR+/HER2+ (0.67 vs 0.52) and HR−/HER2+ BC (0.55 vs 0.41); both methods had a similar PPV overall and in HR−/HER2− tumors. In general, predictive values obtained in the MRI and US group were in line with the results obtained for the MRI group and US group (Supplementary Table 1).

### Correlation in tumor size between imaging and histological assessment

**Fig. 3:**
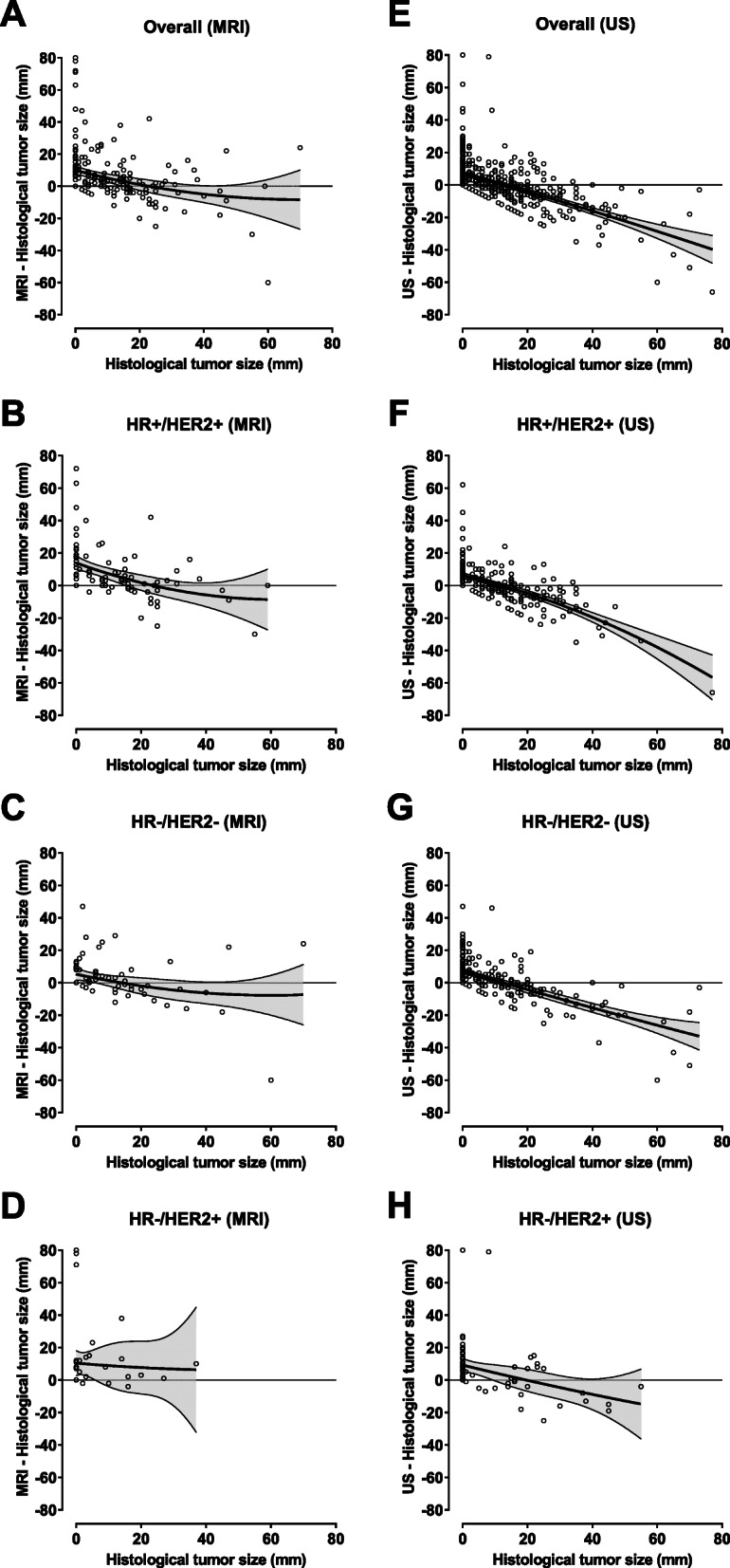
Difference between tumor size according to imaging and residual tumor size versus histological tumor size. Data are shown for all patients with MRI (**a**) and US (**e**) and for HR+/HER2+ (**b**, **f**), HR−/HER2− (**c**, **g**), and HR−/HER2+ tumors (**d**, **h**). Quadratic curve was fitted using the least-squares method; shaded areas represent the 95% confidence interval for fitted curves

Spearman correlation coefficients between pathological tumor size and measurements by MRI and US were 0.55 and 0.51 for all tumors, 0.43 and 0.51 for HR+/HER2+, 0.67 and 0.53 for HR−/HER2−, and 0.61 and 0.43 for HR−/HER2+ BC. Slightly lower correlation coefficients were obtained in patients with both imaging measurements (Supplementary Figure 2). Both MRI and US underestimated and overestimated histological tumor size (Fig. 3, Supplementary Figure 3). There was a tendency towards underestimating tumor size in larger tumors which appeared to be more pronounced for US than for MRI.

## Discussion

The assessment of tumor response to NAT is of importance when planning surgery. In this study, we investigated post-NAT MRI and US for the prediction of pCR and analyzed the accuracy of these methods in the determination of residual tumor size. We found that MRI more often correctly predicted pCR in HR−/HER2+ followed by HR+/HER2+ than in HR−/HER2− tumors. Conversely, US more frequently correctly predicted pCR in HR−/HER2− tumors than in HR+/HER2+ and HR−/HER2+ BC, thus corroborating previously published results [15]. This suggests that MRI may less reliably identify pCR in HR−/HER2− tumors and that assessment of tumor response to NAT should rather be performed by US in this BC subtype. In contrast, Gampenrieder and colleagues reported that MRI correctly predicted pCR more often in HR−/HER2− and HR−/HER2+ tumors than in HR+/HER2+ BC [16]. Moreover, Scheel et al. found no impact of BC subtype on the prediction of pCR by MRI in the ACRIN 6657/I-SPY trial [17].

In our study, residual disease was correctly predicted by US in 77% of HR+/HER2+ and 75% of HR−/HER2− tumors; however, this approach identified only 55% of the cases of non-pCR in HR−/HER2+ BC. In contrast, MRI consistently displayed a high accuracy for the prediction of residual tumor presence across all BC subtypes analyzed (81–86%) with the highest value obtained in HR−/HER2+ tumors. This indicates that MRI misses fewer cases of non-pCR than US and appears to be a method of choice particularly in HR−/HER2+ BC. Nevertheless, other studies demonstrated a variable accuracy of MRI and US for residual disease prediction. For example, both US and MRI were shown to be more accurate for non-pCR prediction in HR+ than in HR− tumors [18]. Furthermore, Gampenrieder et al. found that MRI correctly predicted non-pCR much less frequently in HR−/HER2+ than in HR+/HER2+ and HR−/HER2− tumors [16]. However, the number of patients with HR−/HER2+ tumors was low in that study and in our analysis, which could impact the relative differences in predictive values between this and other BC subtypes.

The evidence regarding the optimal imaging method (MRI versus US) for the prediction of residual tumor size after NAT is conflicting. Studies investigating the correlation between tumor size by imaging and by histology have reported discrepant results with some showing a higher accuracy for MRI vs US [4, 8, 19] and others demonstrating a similar performance of both methods [6, 18, 20]. In our study, the correlation between imaging measurement and final pathology size was similar in the analysis of all tumors. However, we found that US measurements correlated with residual tumor size more closely than MRI in HR+/HER2+ tumors, whereas better correlation coefficients were obtained with MRI than with US in HR−/HER2+ and particularly in HR−/HER2− BC. Our results thus corroborate available evidence suggesting that the correlation between MRI-measured and histological tumor size is highest in HR−/HER2− tumors [21, 22]. However, in a study by Scheel et al., the correlation between tumor size estimated by MRI and final pathology measurement was not affected by BC subtype [17]. Previously, MRI was shown to overestimate and US to underestimate residual tumor size [6] while in other analyses, both methods demonstrated a similar degree of overestimation [23]. According to the NPV values obtained in our study, MRI more often than US correctly predicted the presence of residual tumors measuring 0–10 mm in HR+/HER2+ and HR−/HER2+ tumors. Conversely, US was superior to MRI in terms of correctly estimating the tumor size in lesions measuring > 10 mm in these BC subtypes (as demonstrated by PPV values). Therefore, our results imply that MRI confers a lower risk of underestimating while US is less likely to overestimate residual tumor size in HR+/HER2+ and HR−/HER2+ BC. Compared to our study, Vriens et al. reported slightly higher risks of underestimation and lower probability of overestimation of residual tumor size by MRI and US. They reported that MRI and US were less likely to underestimate lesion size in HR− than in HR+ tumors measuring 0–10 mm (with results favoring US over MRI in this BC subtype, [18]). Conversely, the size of HR+ tumors in that study was less often overestimated than in HR− BC, particularly by US. Evaluation of residual disease by MRI was previously shown to depend on tumor phenotype with a lower rate of underestimation in solid tumors positive for HER2, negative for HR, and triple-negative subtype compared to HR+/HER2− tumors that frequently present as non-mass/diffuse enhancement [24, 25]. Moreover, the extent of the response to NAT was proposed to affect the rates of overestimation by MRI [24]. The presence of enhancing tissue could be misinterpreted as a residual disease, particularly in HR−/HER2− tumors with fibrosis and inflammation-induced during the response to neoadjuvant chemotherapy thus leading to size overestimation. Furthermore, treatment type could impact the imaging accuracy. For example, MRI may less accurately predict pCR after taxane-based therapy due to reduction of contrast enhancement [26]. This limitation should be taken into consideration in assessing the predictive value of MRI in HR−/HER2− tumors. Moreover, given the heterogeneity of NAT regimens in our combined analysis of three substudies, the findings attributed to tumor subtypes may in fact be at least partly attributable to the therapy administered. The NPV values obtained here suggest that MRI and US may underestimate the size of tumors measuring 0–10 mm in 8% and 17% of cases, respectively. However, the PPV values indicate that the risk of tumor size overestimation by MRI and US is far greater and may affect 48% and 39% of cases, respectively. The highest risk of tumor size overestimation was observed in HR−/HER2+ BC in which as much as 59% and 45% of tumors may be smaller in pathologic examination than on MRI and US, respectively. Inaccurate estimation of residual tumor size has implications for the success of the surgery. On the one hand, overestimation may result in excessive resection leading to poor cosmetic outcomes or even the decision for mastectomy instead of breast-conserving surgery. On the other hand, underestimation may lead to excision with tumor-positive margins which may require additional surgery or have a negative impact on long-term outcomes.

Our study has some limitations. First, although the US was mandatory for tumor evaluation, not all patients could be included in this analysis due to missing data. Moreover, both MRI and US were only performed in 174/662 patients which could influence the relative value of these techniques for the prediction of pCR and residual tumor size. Furthermore, although the central reading radiologists were blinded to the US results, the study protocol did not prespecify that the gynecologists should have been blinded to MRI results. Additionally, the quality of MRI image interpretation was ensured by specialized central radiologists, however, the US was performed and interpreted by site gynecologists. Lastly, our study did not provide an insight into the impact of accuracy in the prediction of residual tumor size on successful breast conservation and unnecessary mastectomy rates.

## Conclusions

Our study demonstrated that US and MRI were similarly accurate in predicting the presence of residual tumor after NAT in HR+/HER2+ and HR−/HER2− BC while MRI was more predictive in HR−/HER2+ tumors. The size of HR+/HER2+ and HR−/HER2+ tumors was less likely to be underestimated by MRI while US conferred a lower risk of overestimation in these BC subtypes. The risk of underestimating the size of HR−/HER2− tumors was similarly low for both MRI and US. However, both methods were prone to overestimate the size of each BC subtype, and particularly in HR−/HER2+ tumors. Our findings are clinically relevant for selecting the optimal imaging modality, interpretation of imaging results, and subsequent planning of the surgery in patients with incomplete imaging response to NAT.

## Supplementary Information

**Additional file 1: Table S1.** Prediction of pCR and residual tumor size in MRI and US group.

**Additional file 2: Figure S1.** ROC curves for detecting pCR by MRI and US among patients with both imaging assessments. Data are shown for all tumors (A) and for patients with HR+/HER2+ (B), HR-/HER2- (C) and HR-/HER2+ (D) tumors.

**Additional file 3: Figure S2.** Correlation between tumor size by imaging and pathological tumor size according to breast cancer subtype. Data are shown for tumor size estimations by (A) MRI in all patients with MRI, (B) US in all patients with US, (C) MRI and (D) US in patients with both MRI and US.

**Additional file 4: Figure S3.** Difference between tumor size according to imaging and residual tumor size versus residual tumor size. Data are shown for all patients with both MRI and US (A) and for HR+/HER2+ (B), HR-/HER2- (C) and HR-/HER2+ tumors (D). Quadratic curve was fitted using the least squares method.

## Data Availability

Data used for this analysis are available upon reasonable request to the corresponding author.

## Abbreviations

BC: Breast cancer
EoT: End of treatment
ET: Endocrine therapy
MRI: Magnetic resonance imaging
NAT: Neoadjuvant therapy
NPV: Negative predictive value
pCR: Pathological complete response
PPV: Positive predictive value
T-DM1: Trastuzumab emtansine
TN: Triple-negative
US: Ultrasound
