# Supplementary material for: Magnetic resonance imaging and ultrasound for prediction of residual tumor size in early breast cancer within the ADAPT subtrials
Source: Breast Cancer Res. 2021 Mar 18;23:36. doi: 10.1186/s13058-021-01413-y (PMC7977310; doi:10.1186/s13058-021-01413-y)
Supplement: Supplementary file 1 — Additional file 1: Table S1. Prediction of pCR and residual tumor size in MRI and US group. [file 13058_2021_1413_MOESM1_ESM.docx]

Supplementary Table 1: Prediction of pCR and residual tumor size in MRI and US group.

| **Group** |  | **pCR,**  **N** | **CR,**  **N** | **TN, N** | **FP, N** | **TP, N** | **FN, N** | **Specificity (CL)** | **Sensitivity (CL)** | **NPV**  **(CL)** | **PPV**  **(CL)** |
| --- | --- | --- | --- | --- | --- | --- | --- | --- | --- | --- | --- |
| **Overall** | **MRI**  **(N=171)** | 64 | 60 | 42 | 22 | 89 | 18 | 0.66  (0.53-0.77) | 0.83  (0.75-0.90) | 0.70  (0.57-0.81) | 0.80  (0.72-0.87) |
|  | **US**  **(N=171)** | 64 | 46 | 32 | 32 | 93 | 14 | 0.50  (0.37-0.63) | 0.87  (0.79-0.93) | 0.70  (0.54-0.82) | 0.74  (0.66-0.82) |
| **HR+/HER2+** | **MRI**  **(N=69)** | 24 | 16 | 12 | 12 | 41 | 4 | 0.50  (0.29-0.71) | 0.91  (0.79-0.98) | 0.75  (0.48-0.93) | 0.77  (0.64-0.88) |
|  | **US**  **(N=69)** | 24 | 17 | 14 | 10 | 42 | 3 | 0.58  (0.37-0.78) | 0.93  (0.82-0.99) | 0.82  (0.57-0.96) | 0.81  (0.68-0.90) |
| **HR-/HER2-** | **MRI**  **(N=68)** | 24 | 32 | 18 | 6 | 30 | 14 | 0.75  (0.53-0.90) | 0.68  (0.52-0.81) | 0.56  (0.38-0.74) | 0.83  (0.67-0.94) |
|  | **US**  **(N=68)** | 24 | 18 | 10 | 14 | 36 | 8 | 0.42  (0.22-0.63) | 0.82  (0.67-0.92) | 0.56  (0.31-0.79) | 0.72  (0.58-0.84) |
| **HR-/HER2+** | **MRI**  **(N=34)** | 16 | 12 | 12 | 4 | 18 | 0 | 0.75  (0.48-0.93) | 1  (0.82-1) | 1  (0.74-1) | 0.82  (0.60-0.95) |
|  | **US**  **(N=34)** | 16 | 11 | 8 | 8 | 15 | 3 | 0.50  (0.25-0.75) | 0.83  (0.59-0.96) | 0.73  (0.39-0.94) | 0.65  (0.43-0.84) |
| **Group** |  | **≤10 mm, N** | **≤10 mm, N** | **TN, N** | **FP, N** | **TP, N** | **FN, N** | **Specificity (CL)** | **Sensitivity (CL)** | **NPV**  **(CL)** | **PPV**  **(CL)** |
| **Overall** | **MRI**  **(N=151)** | 100 | 69 | 62 | 38 | 44 | 7 | 0.62  (0.52-0.72) | 0.86  (0.74-0.94) | 0.90  (0.80-0.96) | 0.54  (0.42-0.65) |
|  | **US**  **(N=169)** | 112 | 94 | 83 | 29 | 46 | 11 | 0.74  (0.65-0.82) | 0.81  (0.68-0.90) | 0.88  (0.80-0.94) | 0.61  (0.49-0.72) |
| **HR+/HER2+** | **MRI**  **(N=67)** | 39 | 18 | 16 | 23 | 26 | 2 | 0.41  (0.26-0.58) | 0.93  (0.77-0.99) | 0.89  (0.65-0.99) | 0.53  (0.38-0.68) |
|  | **US**  **(N=70)** | 41 | 36 | 31 | 10 | 24 | 5 | 0.76  (0.60-0.88) | 0.83  (0.64-0.94) | 0.86  (0.71-0.95) | 0.71  (0.53-0.85) |
| **HR-/HER2-** | **MRI**  **(N=58)** | 40 | 36 | 31 | 9 | 13 | 5 | 0.78  (0.62-0.89) | 0.72  (0.47-0.90) | 0.86  (0.71-0.95) | 0.59  (0.36-0.79) |
|  | **US**  **(N=68)** | 47 | 36 | 31 | 16 | 16 | 5 | 0.66  (0.51-0.79) | 0.76  (0.53-0.92) | 0.86  (0.71-0.95) | 0.50  (0.32-0.68) |
| **HR-/HER2+** | **MRI**  **(N=26)** | 21 | 16 | 15 | 6 | 5 | 0 | 0.71  (0.48-0.89) | 1  (0.48-1) | 1  (0.78-1) | 0.46  (0.17-0.77) |
|  | **US**  **(N=31)** | 24 | 22 | 21 | 3 | 6 | 1 | 0.88  (0.68-0.97) | 0.86  (0.42-1) | 0.96  (0.77-1) | 0.67  (0.30-0.93) |

TP, true positive examination; FP, false positive examination; TN, true negative examination and FN; false negative examination. For pCR, TN was defined as CR at imaging and pCR, FP as no CR at imaging and pCR, TP as no CR at imaging and no pCR, and FN as CR at imaging and no pCR. For residual tumor at histology, TN was defined as tumor size of ≤10 mm by imaging and equivalent tumor size at histology, FP was defined as tumor size of >10 mm by imaging and tumor size of ≤10 mm at histology; TP was defined as tumor size of >10 mm by imaging and equivalent tumor size at histology, and FN was defined as tumor size of ≤10 mm by imaging and tumor size of >10 mm at histology. PPV, Positive Predictive Value, defined as P(P=1|I=1); NPV, Negative Predictive Value, defined as P(P=0|I=0); SENS, Sensitivity, defined as P(I=1|P=1); SPEC, Specificity, defined as P(I=0|P=0 where P(A|B) denotes the conditional probability of event A given that event B has occurred. P=1 denotes a residual invasive tumor size of >10 mm at histology and I=1 denotes tumor size of >10 mm at imaging; CL, exact 95% confidence limits (Clopper-Pearson).
