# Supplementary material for: Magnetic resonance imaging and ultrasound for prediction of residual tumor size in early breast cancer within the ADAPT subtrials
Source: Breast Cancer Res. 2021 Mar 18;23:36. doi: 10.1186/s13058-021-01413-y (PMC7977310; doi:10.1186/s13058-021-01413-y)
Supplement: Supplementary file 2 — Additional file 2: Figure S1. ROC curves for detecting pCR by MRI and US among patients with both imaging assessments. Data are shown for all tumors (A) and for patients with HR+/HER2+ (B), HR-/HER2- (C) and HR-/HER2+ (D) tumors. [file 13058_2021_1413_MOESM2_ESM.docx]

Supplementary Figure 1. ROC curves for detecting pCR by MRI and US among patients with both imaging assessments. Data are shown for all tumors (A) and for patients with HR+/HER2+ (B), HR-/HER2- (C) and HR-/HER2+ (D) tumors.
