# Supplementary material for: Magnetic resonance imaging and ultrasound for prediction of residual tumor size in early breast cancer within the ADAPT subtrials
Source: Breast Cancer Res. 2021 Mar 18;23:36. doi: 10.1186/s13058-021-01413-y (PMC7977310; doi:10.1186/s13058-021-01413-y)
Supplement: Supplementary file 3 — Additional file 3: Figure S2. Correlation between tumor size by imaging and pathological tumor size according to breast cancer subtype. Data are shown for tumor size estimations by (A) MRI in all patients with MRI, (B) US in all patients with US, (C) MRI and (D) US in patients with both MRI and US. [file 13058_2021_1413_MOESM3_ESM.docx]

Supplementary Figure 2. Correlation between tumor size by imaging and pathological tumor size according to breast cancer subtype. Data are shown for tumor size estimations by (A) MRI in all patients with MRI, (B) US in all patients with US, (C) MRI and (D) US in patients with both MRI and US.
