# Supplementary material for: Magnetic resonance imaging and ultrasound for prediction of residual tumor size in early breast cancer within the ADAPT subtrials
Source: Breast Cancer Res. 2021 Mar 18;23:36. doi: 10.1186/s13058-021-01413-y (PMC7977310; doi:10.1186/s13058-021-01413-y)
Supplement: Supplementary file 4 — Additional file 4: Figure S3. Difference between tumor size according to imaging and residual tumor size versus residual tumor size. Data are shown for all patients with both MRI and US (A) and for HR+/HER2+ (B), HR-/HER2- (C) and HR-/HER2+ tumors (D). Quadratic curve was fitted using the least squares method. [file 13058_2021_1413_MOESM4_ESM.docx]

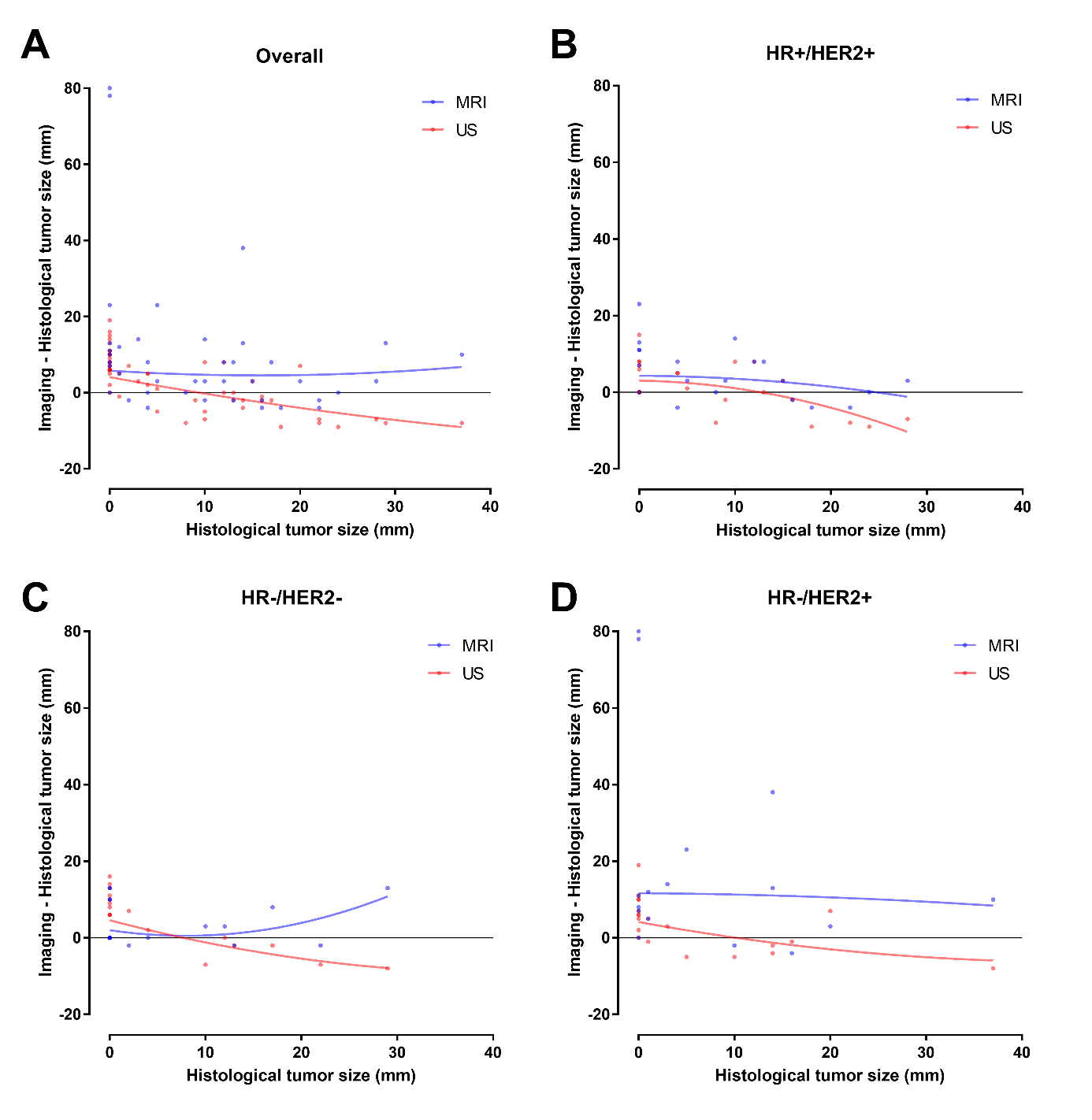


Supplementary Figure 3. Difference between tumor size according to imaging and residual tumor size versus residual tumor size. Data are shown for all patients with both MRI and US (A) and for HR+/HER2+ (B), HR-/HER2- (C) and HR-/HER2+ tumors (D). Quadratic curve was fitted using the least squares method.
